# Unravelling technical domain barriers and non-technical skill barriers among interprofessional teams during in-hospital cardiac arrest: a questionnaire-based survey

**DOI:** 10.1186/s12245-026-01224-y

**Published:** 2026-04-13

**Authors:** Prabha Prakash, Kirtana Raghurama Nayak, Elsa Sanatombi Devi, Souvik Chaudhuri, Abraham Samuel Babu, Dinker Ramananda Pai, Vimal Krishnan S

**Affiliations:** 1 Department of Emergency Medicine, Kasturba Medical College, Manipal Academy of Higher Education, Manipal, India; 2 Department of Physiology, Kasturba Medical College, Manipal Academy of Higher Education, Manipal, India; 3 Department of Medical Surgery, Manipal College of Nursing, Manipal Academy of Higher Education, Manipal, India; 4 Department of Critical Care Medicine, Kasturba Medical College, Manipal Academy of Higher Education, Manipal, India; 5 Department of Physiotherapy, Manipal College of Health Professions, Manipal Academy of Higher Education, Manipal, India; 6 Department of Surgery, Mahatma Gandhi Medical College and Research Institute, Sri Balaji Vidyapeeth, Puducherry, India

**Keywords:** Barriers, Interprofessional team, Cardiopulmonary resuscitation, Technical skills, Non-technical skills

## Abstract

**Background:**

In spite of focused cardiopulmonary resuscitation (CPR) training, the in-hospital cardio-pulmonary resuscitation (CPR) performance often remains suboptimal, due to technical domain barriers and non-technical skill (NTS) barriers. We aimed to determine the technical domain and NTS barriers encountered by the in-hospital interprofessional (IP) CPR resuscitation teams through a cross-sectional, quantitative, analytical survey, and to determine whether a poor NTS score alone is associated with non-adherence to high-quality CPR.

**Methods:**

A validated questionnaire was developed comprising 17 items on technical domain barriers and 9 on the NTS barrier domain. A web-based Likert-scale questionnaire was administered to assess perception-based barriers among 400 IP team members across healthcare settings in India, focusing on technical domain barriers and NTS domain barriers. IP members with at least 1 year of work experience, hands-on CPR training, and in-hospital CPR experience were recruited.

**Results:**

There were 32.75% doctors, 29.75% nurses, 24% respiratory therapists, 10.25% emergency technicians, and 2.25% ICU technicians. The logistic regression analysis revealed that only five items independently predicted a poor technical domain score: inability to identify cardiac rhythm, non-working defibrillator, unavailability of a supraglottic airway device, unavailability of a correct-size laryngoscope blade, and a delay in loading medications. Five NTS items independently predicted a poor NTS score: lack of clear instructions from the team leader, lack of awareness of the dynamic nature of resuscitation, lack of closed-loop communication, lack of assigned tasks completed by a team member, and lack of knowledge sharing among team members. A poor NTS score is associated with perceived non-adherence to high-quality CPR (Chi-Square test, p-value < 0.001).

**Conclusion:**

Both NTS barriers and technical domain barriers were associated with perceived non-adherence to high-quality CPR.

**Trial registration:**

Clinical Trial Registry of India CTRI/2025/05/086727 registered on 13/05/2025.

**Supplementary information:**

The online version contains supplementary material available at 10.1186/s12245-026-01224-y.

## Introduction

In-hospital cardiac arrest (IHCA) has an incidence of about 23.4 per 1000 hospital admissions in low-middle income countries [1–3]. Guideline-based resuscitation care and an effective response system are crucial for improvement in IHCA outcomes [1]. Adherence to the American Heart Association (AHA) guidelines for high-quality cardiopulmonary resuscitation (CPR) improves survival outcomes [4]. Deficiencies among CPR providers in non-technical skills (NTS) lead to a higher mortality during IHCA [5]. Likewise, in the technical domain, appropriate technical skills (TS) and optimal equipment are essential for ensuring high-quality CPR [6–8]. Despite CPR training, literature reveals that CPR performance often remains sub-optimal, with poor outcomes [9]. Compliance with CPR guidelines is about 86% in monitored hospital areas but falls to 67% in unmonitored wards [5]. The absence of a unified national in-hospital cardiac arrest registry impedes the recognition of barriers and hinders continuous improvement in the technical domain and NTS among CPR providers [10]. Among NTS components, ineffective communication and poor team coordination have been identified as major contributors to medical errors during high-stress situations.

 [11]. Thus, we need to integrate NTS training to enhance CPR team performance, as emphasized in the 2025 AHA guidelines, which also propose competency upon course completion [12].

This study aimed to determine the NTS, and technical domain barriers encountered by the.

in-hospital CPR providers. The primary objective was to determine the specific NTS, and technical domain barriers encountered by the IP in-hospital CPR providers during CPR scenarios that independently predict poor NTS and technical domain scores. The secondary objective was to evaluate whether a poor NTS score is associated with perceived non-adherence to high-quality CPR.

## Methods

This study is an analytical cross-sectional survey of IP CPR providers, including doctors, respiratory therapists, nurses, and emergency technicians. The survey was conducted from June 2025 to October 2025 across India after institutional ethical clearance (IEC1: 107/2025) and CTRI registration (CTRI/2025/05/086727). The reporting of the manuscript has been done by following the Consensus-Based Checklist for Reporting of Survey Studies CROSS Checklist) [13] and the Checklist for Reporting Results of Internet E-Surveys (CHERRIES) [14].

### Sample size

The sample size was estimated using Slovin’s formula for proportion-based surveys based on previous studies [15]. We used Slovin’s formula as a pragmatic approach to sample size estimation, based on the approximate number of IP healthcare providers in India involved in CPR.

$${\mathrm{Sample}}\,{\mathrm{size}}\,\left( {\mathrm{n}} \right)\,{\text{ = }}\,{\mathrm{N}}\,{\mathrm{/}}\,\left( {{\mathrm{1}}\,{\text{ + }}\,{\text{N }}{{\mathrm{e}}^{\mathrm{2}}}} \right)$$

where n is the required sample, N is the total population of professionals who are likely to be involved in providing in-hospital CPR, and e is the margin of error. The sampling frame (N) included emergency medicine physicians and nurses, intensive care unit physicians and nurses, and respiratory therapists across India. Based on data from the Society for Emergency Medicine India, the Indian Society of Critical Care Medicine, the National Sample Survey Office on nurses, and the Indian Association of Respiratory Care, the total number of CPR providers in India was estimated at approximately 57,500 [16–19]. Using a 5% margin of error (e = 0.05), the required sample size was 398, rounded to 400.

We considered a non-response rate of 20% (d = 0.20). The adjusted target sample (N₁) was calculated as N₁ = n/(1 − d), yielding approximately 500 IP CPR providers to whom the questionnaire should be sent.

Inclusion criteria:

- Interprofessional team members who had received hands-on CPR training and had at least one year of clinical experience with exposure to IHCA resuscitation, either as active participants or as observers.
- Registered doctors, emergency physicians, intensivists, nurses, respiratory therapists, and emergency medical technicians.

Exclusion criteria:

- Refusal to participate or those who did not complete the survey.

### Sampling method

A nationwide web-based cross-sectional survey was conducted to identify barriers to In-hospital CPR encountered by healthcare professionals. The questionnaire was disseminated electronically via professional networks and institutional contacts at multiple healthcare institutions across different regions of India. A convenience sampling strategy was selected, in which voluntary respondents who completed the online survey were included until the target sample size of 400 participants was reached. Respondents were healthcare professionals from various institutions across the country.

Data collection invitation: A self-administered, web-based questionnaire was used after consent to participate. The SurveyMonkey survey link (SurveyMonkey Inc., San Mateo, CA, USA) was distributed nationwide through multiple professional contacts and platforms, as well as professional WhatsApp groups. The SurveyMonkey link allowed only a single response per participant, thereby preventing duplicate submissions. The questionnaire was designed to capture healthcare professionals’ perceptions and self-reported experiences regarding barriers to high-quality CPR, based on their recollection of resuscitation events encountered in routine clinical practice over the preceding year. Participants completed the survey independently, as the researcher was not present during data collection. This approach was adopted to minimise interviewer and social desirability bias and to ensure anonymity and confidentiality. This also provided respondents the flexibility to complete the survey at their own convenience.

### Development of a data collection tool

Data were collected using a novel, structured questionnaire developed by the research team through a comprehensive review of existing CPR and IHCA literature. The questionnaire items underwent one round of content validation by ten IP domain experts (emergency medicine physicians, intensive care physicians, respiratory therapists, and emergency and critical care nurses) to ensure clarity, relevance, and construct alignment. Quantitatively, expert suggestions were measured using Lawshe’s content validity ratio (CVR). Individual experts had provided scores on each item independently based on relevant (2), somewhat relevant (1), and non-relevant (0) scale on a three- point Likert scale. The initial 56-item questionnaire was reduced to 31 items following content validation (17 items on technical domain barriers, 9 on NTS, and 5 on logistics barriers). The technical domain encompassed both procedural skill-related competencies and equipment-related factors that are relevant to high-quality CPR. The scale-level CVR (S-CVR) was 1.00, indicating agreement among the ten experts. These findings confirmed that the questionnaire items coherently measured their respective constructs and were appropriate. The questionnaire was pilot-tested among 10 healthcare professionals to assess clarity, feasibility, and completion time. Subsequent reliability testing demonstrated excellent internal consistency across all domains, with Cronbach’s alpha values of 0.923 for Technical Skills, 0.950 for Non-Technical Skills, and 0.853 for Logistics.

### Questionnaire-based assessment of CPR to the success of the interprofessional team

Information regarding the barriers encountered during IHCA resuscitation was collected using a structured, self-administered questionnaire. The complete questionnaire is provided as Supplementary Material (Supplementary or Additional File 1). The survey instrument consisted of 40 items; each was rated on a five-point Likert scale based on how frequently the participant experienced that barrier in the past year.

We intentionally chose a one-year recall window to minimize recall bias, especially for perception-based survey items. Longer recall periods may introduce inaccuracies due to memory decay, especially for emotionally intense or infrequent events, such as mortality [20]. We acknowledge that a shorter recall period could reduce the number of events respondents consider when answering the questionnaire, especially in low-volume settings. To address this, we would like to clarify that respondents were not asked to quantify exact frequencies, but rather to rate how often they encountered specific barriers relative to the resuscitations they personally experienced in the past year.

Importantly, the perception of frequency was more strongly influenced by proportion than by absolute count. Thus, the Likert responses reflect subjective proportional experience, which is meaningful in understanding perceptions across diverse healthcare environments.

The survey response options for NTS barriers and technical domain barriers were based on perceptions of the barriers and included: never (1), rarely (2), sometimes (3), often (4), and very often (5). The survey included items exploring perceived barriers in the NTS domain (including communication, leadership, and situational awareness) and technical domain aspects of CPR (technical skills and equipment). The responses reflected self-reported perceptions of barriers rather than objective assessments of individual performance. Higher scores indicated more frequent encounters with the respective barrier. The questionnaire had four domains. Demographic characteristics (9 items), technical domain barriers encompassing technical skills and equipment (17 items), NTS barriers (9 items), and logistics barriers (5 items). All items were scored in the same direction, except for one reverse-coded question related to post-resuscitation debriefing, which was not a component of any barrier domain. For this item, responses of never or rarely indicated a negative practice, and therefore represented a higher barrier despite being scored lower on the Likert scale. Appropriate reverse-coding was applied during statistical analysis.

Perceived adherence to high-quality CPR was assessed using questionnaire items based on the components of high-quality CPR recommended by the American Heart Association (AHA) guidelines [21]. These components were explicitly listed in the questionnaire to guide respondents’ responses. The components listed in the questionnaire were:

- Start compressions within 10 s after recognising cardiac arrest.
- Chest compression rate – 100–120 compressions per minute.
- Chest compression depth: At least 5 cm for adults.
- Chest compression fraction > 80%.
- Allow the chest to completely recoil after each compression.
- Minimize interruptions in compressions to < 10 s.
- Give effective breaths to ensure a visible chest rise.

Perceived adherence to high-quality CPR was only when all the components listed above were followed, without a single omission. Omission of a single component listed above was perceived as non-adherence to high-quality CPR.

### Statistical analysis

The data were analyzed using IBM SPSS Statistics for Windows, version 6.0 (Armonk, NY: IBM). The basic demographic variable was expressed as frequencies and percentages. Normality of distribution was assessed using the Shapiro-Wilk test and Q-Q plots. For both the NTS and technical domains, the scores from all the questions were added. A total domain score ≥ 75th percentile was considered a “poor” score, while scores below this threshold were considered “adequate” scores.

In the absence of established absolute cut-offs for non-technical skills (NTS) and technical domain scores, we used a percentile-based classification approach. Scores ≥ 75th percentile were defined as a poor score, representing the highest quartile of the distribution. This approach was used for survey-based and ordinal data, where distribution-driven thresholds provide an objective, reproducible means of categorization. Barua A et al. and Mulla M et al. supported the use of percentile-based decision rules for interpreting ordinal variables in the absence of predefined benchmarks, emphasizing their validity in health research. They utilized the upper quartile (≥ 75th percentile) as a pragmatic threshold to delineate higher-risk or poorer-performing subgroups in studies [22–23].

The mean ± standard deviation (SD) and median (inter-quartile range [IQR]) were calculated. The questionnaire items’ reliability was determined using Cronbach’s alpha. For the respective NTS and technical domains, univariate analyses to predict a poor score were performed for each survey question. For the questions found significant in the univariate analysis as predictors of poor NTS and technical domain scores, a logistic regression was built to identify independent predictors, with adjusted odds ratios (aOR) and 95% confidence intervals (CIs). Multicollinearity was assessed by examining Variance Inflation Factors (VIFs) and condition indices, with cut-offs of 5 for VIF and 10 for condition index. The Benjamini–Hochberg (BH) procedure was applied to control the false discovery rate associated with multiple hypothesis testing. The variables that had a p-value < BH critical p-values were determined as independent predictors of poor NTS and technical domain scores. For tests of association between the categorical variables, the Chi-Square test and Fisher’s exact test were used. The association between poor NTS and poor technical domain scores, with a lack of complete perceived non-adherence to the high-quality CPR, was analyzed using the Chi-Square test or Fisher’s exact test, and a p-value < 0.05 was considered significant. Only complete responses were included in the final analysis; therefore, no missing data were present.

## Results

The survey link was shared with 500 eligible professionals; 498 responses were received, of which 98 were incomplete. A final sample of 400 complete responses was included in the analysis.

**Fig. 1:**
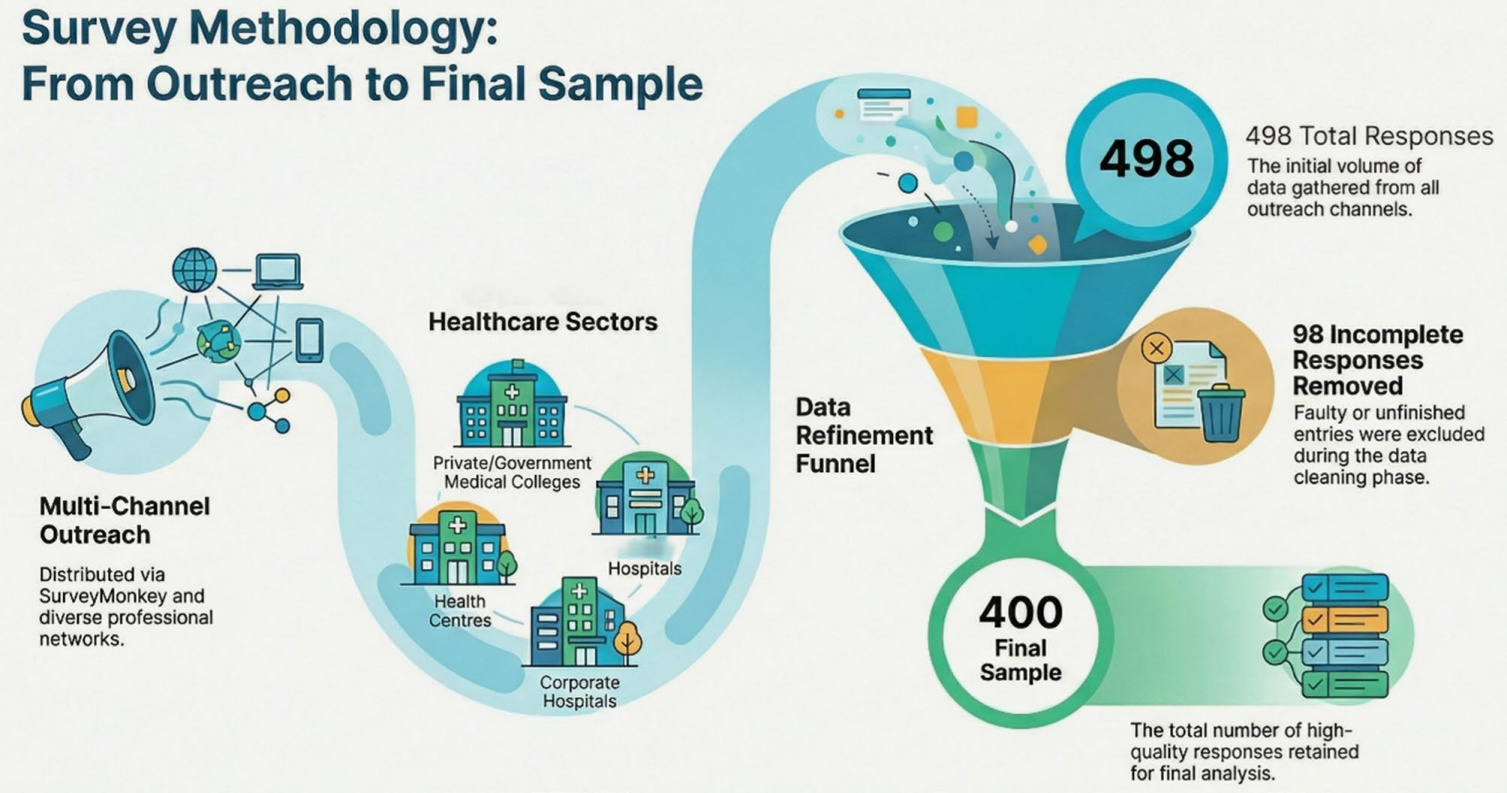
The survey methodology for achieving 400 survey responses. The authors used Google NotebookLM to generate the infographic in Fig. 1 based on concepts developed by the authors. All outputs were reviewed and verified by the authors

There were 400 IP participants; the demographic characteristics of the IP team are depicted in Table 1. The details of the participants’ recruitment are shown in Fig. 1. The responses from participants to the cardiopulmonary resuscitation barriers questionnaire, faced in the past year, in the technical domain, non-technical skills, and logistics domains, are shown in Table 2. The NTS domain scores were summed up to 45 points from nine items (median score 19.50; IQR [12–28]), and the technical domain scores were summed up to a total of 85 points (median score 37; IQR [30–50]).

**Table 1:** Demographic characteristics of the interprofessional healthcare providers who participated in the survey

S.No	Demographic variables	Category	*N* = 400 (%)
01.	Gender	Male	175(43.75%)
		Female	225(56.25%)
02.	Geographical zone	North	43(10.75%)
		South	286(71.50%)
		East	51(12.75%)
		West	12(3%)
		Central	8(2%)
03.	Type of institution	Government District hospital/CHC/PHC	30(7.50%)
		Government Medical College	34(8.50%)
		Private Medical College	210(52.50%)
		Private Hospital	99(24.75%)
		Nursing Home	6(1.50%)
		Mission hospitals	4(1.00%)
		Others	17(4.25%)
04.	Interprofessional teams	Doctors	131(32.75%)
		Nurse	119(29.75%)
		Respiratory Therapist	96(24%)
		Emergency Medicine Technician	41(10.25%)
		ICU Technician	9(2.25%)
		MBBS Intern	2(0.50%)
		Cardiovascular Technologist	2(0.50%)
05	Working in Rural India	Yes	107(26.75%)
		No	293(73.25%)
06.	AHA-certified BLS providers	Yes	300(75%)
		No	100(25%)
07.	AHA-certified ACLS providers	Yes	276(69%)
		No	124(31%)
08	Ever participated as a team member during CPR	Yes	369(92.25%)
		No	31(7.75%)

Legends: AHA, American Heart Association; BLS, Basic Life Support; ACLS, Advanced Cardiac Life Support; CPR, Cardio-pulmonary resuscitation; ICU, Intensive care unit; CHC, Community health center; PHC, Primary health center; MBBS, Bachelor of Medicine, Bachelor of Surgery

**Table 2:** Responses from the participants to the cardio-pulmonary resuscitation barriers questionnaire faced by them in the past year in the Technical, Non-technical skills, and logistics domains

	Numerical ratings on a scale of 1 to 5 in percentage (from never to most often)				
	Numerical rating 1 (Never encountered) *n*(%)	Numerical rating 2 (Rarely encountered) *n*(%)	Numerical rating 3 (Sometimes encountered) *n*(%)	Numerical rating 4 (Often encountered) *n*(%)	Numerical rating 5 (Very often encountered) *n* (%)
**A. Questions on the Technical domain**					
Delay in initiation of CPR in wards	75 (18.75%)	81 (20.25%)	162 (40.50%)	72 (18.00%)	10 (2.50%)
Delaying the initiation of CPR due to airway contamination (blood/vomitus/secretions)	97(24.25%)	71(17.75%)	109(27.25%)	79(19.75%)	44(11.00%)
Difficulty in identifying shockable or non-shockable rhythm	110(27.50%)	101(25.25%)	120(30.00%)	62(15.50%)	7(1.75%)
Non-working defibrillator during CPR	132(33.00%)	123(30.75%)	73(18.25%)	63(15.75%)	9(2.25%)
Unfamiliarity with a defibrillator model	122(30.50%)	92(23.00%)	107(26.75%)	73(18.25%)	6(1.50%)
Malfunctioning of the suction apparatus	78(19.50%)	80(20.00%)	92(23.00%)	102(25.50)	48(12%)
Unavailability of a bag-valve-mask (BVM)	174(43.50%)	70(17.50%)	84(21.00%)	64(16.00%)	8(2.00%)
Unavailability of a correctly sized oropharyngeal airway	168(42.00%)	89(22.25%)	58(14.50%)	68(17.00%)	17(4.25%)
Unavailability of a supraglottic airway device	157(39.25%)	87(21.75%)	58(14.50%)	79(19.75%)	19(4.75%)
Unavailability of a correctly sized laryngoscope blade	176(44.00%)	83(20.75%)	61(15.25%)	59(14.75%)	21 (5.25%)
Unavailability of a stylet or a bougie during difficult intubation	99(24.75%)	121(30.25%)	89(22.25%)	75(18.75%)	16(4.00%)
Unavailability of end-tidal CO_2_ monitoring	140(35.00%)	64(16.00%)	73(18.25%)	77(19.25%)	46(11.50%)
CPR interruptions > 10 s to secure advance airway	89(22.25%)	115(28.75%)	101(25.25%)	71(17.75%)	24(6.00%)
Delay in loading medications	158(39.50%)	101(25.25%)	67(16.75%)	64(16.00%)	10(2.50%)
Errors in medication administration	220(55.00%)	91(22.75%)	33(8.25%)	50(12.50%)	6(1.50%)
Lack of CCF monitoring	134(33.50%)	75(18.75%)	76(19.00%)	83(20.75%)	32(8.00%)
Lack of a CPR feedback device	128(32.00%)	59(14.75%)	83(20.75%)	90(22.50%)	40(10.00%)
**B. Questions on Non-technical skills domain**					
Lack of Role allocation by the team leader	128(32.00%)	84(21.00%)	78(19.50%)	85(21.25%)	25(6.25%)
Unclear instructions from the team leader	136(34.00%)	95(23.75%)	78(19.50%)	78(19.50%)	13(3.25%)
Lack of awareness of the dynamic nature of resuscitation	125(31.25%)	103(25.75%)	78(19.50%)	78(19.50%)	16(4.00%)
Attention diversion due to other cases	167(41.75%)	93(23.25%)	73(18.25%)	60(15.00%)	7(1.75%)
Lack of closed-loop communication among team members	119(29.75%)	93(23.25%)	88(22.00%)	85(21.25%)	15(3.75%)
Ineffective critical decision-making from the team leader	148(37.00%)	106(26.50%)	76(19.00%)	62(15.50%)	8(2.00%)
Lack of clear vocal summarization of the scenario	129(32.25%)	106(26.50%)	76(19.00%)	69(17.25%)	20(5.00%)
Lack of task completion assigned to the team member	140(35.00%)	108(27.00%)	80(20.00%)	61(15.25%)	11(2.75%)
Lack of knowledge sharing among team members	133(33.25%)	90(22.50%)	85(21.25%)	76(19.00%)	16(4.00%)
**C. Questions on the Logistics domain**					
Absence of AHA-ACLS trained team members	165(41.25%)	75(18.75%)	74(18.50%)	72(18.00%)	14(3.50%)
Participation in a post-resuscitation debriefing after completing CPR	116(29.00%)	84(21.00%)	78(19.50%)	84(21.00%)	38(9.50%)
Difficulty in reaching the CPR location	138(34.50%)	85(21.25%)	80(20.00%)	79(19.75%)	18(4.50%)
Overcrowding of patient relative around the patient	98(24.50%)	67(16.75%)	119(29.75%)	82(20.50%)	34(8.50%)
Perceived non-adherence to high-quality CPR components^*^	46(11.50%)	86(21.50%)	50(12.50%)	150(37.50%)	68(17.00%)

Legend: CPR, Cardio-pulmonary resuscitation; AMBU, Artificial Manual Breathing Unit; CCF, Chest compression fraction; AHA, American Heart Association; ACLS, Advanced cardiac life support

*High-quality CPR, as per the 2020 AHA protocol, includes the following components as listed below (a-g). Thus, perceived adherence to high-quality CPR refers to the perceived adherence to all the components listed below. Perceived non-adherence to high-quality CPR refers to omitting any one of the following components. These components were mentioned in the questionnaire survey and are the following:

a. Start compressions within 10 s after recognising cardiac arrest

b. Chest compression rate – 100–120 compressions per minute

c. Chest compression depth: At least 5 cm for adults

d. Chest compression fraction > 80%

e. Allow the chest to completely recoil after each compression

f. Minimize interruptions in compressions to < 10 s

g. Give effective breaths to ensure a visible chest rise

The NTS score was considered poor if it was ≥ 28 points. The Cronbach’s alpha for NTS was 0.961, indicating good internal consistency. The Chi-Square test of association between each of the items in the NTS domain with a poor NTS score showed that there was a significant association with each of the nine questions and a poor NTS score (Additional file 2). The univariate analysis of the items with poor NTS scores revealed that all 9 items were significant predictors of poor NTS scores. The multivariable logistic regression with BH adjusted p-values showed that five NTS domain items independently predicted a poor NTS score- lack of clear instructions by team leader (aOR 11.02, 95% CI 1.50-80.79, p-value 0.018), lack of awareness of the dynamic nature of resuscitation (aOR 35.60, 95% CI 6.07-208.59, p-value < 0.001), a lack of closed loop communication (aOR 51.11, 95% CI 7.828–336.68, p-value < 0.001), a lack of task completion assigned to a team member (aOR 136.14, 95% CI 6.088–3044.50, p-value 0.02), and a lack of knowledge sharing among the team members (aOR 6.11, 95% CI 1.415–26.36, p-value 0.015) (Table 3). The dynamic nature of resuscitation refers to rapidly changing, time-sensitive conditions such as transitions from shockable to non-shockable rhythms, intravenous line displacement and drug extravasation, or even supraglottic airway dislodgement, as well as variations in end-tidal carbon dioxide levels, which require prompt recognition and intervention by the CPR team.

**Table 3:** Univariate and multivariable logistic regression of the individual items in NTS to predict an overall poor NTS score

Univariate Analysis				Multivariable logistic regression			Benjamini-Hochberg adjusted *p*-value to reduce false discovery rate
Items	P value	Unadjusted OR	95% CI	p-value	Adjusted OR	95% CI	p value < BH value
Lack of role allocation by the team leader	< 0.001	41.59	22.34–77.43	0.77	4.387	0.852–22.586	0.05
**Lack of clear instructions by the team leader**	< 0.001	112.928	49.275-258.806	**0.018**	11.018	1.503–80.792	**0.03**
**Lack of awareness of the dynamic nature of resuscitation**	< 0.001	132.272	57.108-306.362	**< 0.001**	35.608	6.079-208.595	**0.01**
Lack of focused attention diversion	< 0.001	60.365	24.725-147.378	0.969	1.052	0.082–13.550	0.06
**Lack of closed-loop communication**	< 0.001	97.429	46.084-205.977	**< 0.001**	51.108	7.828-336.682	**0.01**
Lack of critical decision-making by the team leader	< 0.001	110.128	38.196-317.524	0.053	10.045	0.972–103.790	0.04
Lack of clear vocal summarization among team members	< 0.001	102.335	44.908-233.198	0.051	5.223	0.996–27.393	0.03
**Lack of task completion assigned to the team member**	< 0.001	541.842	73.149-4013.618	**0.002**	136.140	6.088- 3044.502	**0.02**
**Lack of knowledge sharing among team member**	< 0.001	143.690	59.833-345.077	**0.015**	6.107	1.415–26.355	**0.02**

Legends: NTS, Non-technical skills; OR, Odd’s ratio; CI, confidence Interval; BH value, Benjamini-Hochberg value

**Table 4:** Univariate and multivariable logistic regression of items in the technical domain to predict an overall poor technical domain score

Univariate Analysis				Multivariable logistic regression			Benjamini Hochberg adjusted *p*-value to reduce FDR
Items	P value	Unadjusted OR	95% CI	p-value	Adjusted OR	95% CI	(p value < BH value)
**Inability to identify cardiac rhythm**	< 0.001	59.32	25.49-138.05	**< 0.001**	26.888	3.597-201.015	**0.01**
**Non-working defibrillator**	< 0.001	41.09	19.59–86.21	**0.008**	15.199	2.024-114.116	**0.01**
Unfamiliarity with different models of defibrillators	< 0.001	74.91	33.23-168.87	0.037	5.942	1.109–31.837	0.03
Malfunctioning of the suction apparatus	< 0.001	10.28	6.10-17.35	0.021	5.055	1.281–19.944	0.02
Unavailability of a bag-valve-mask device	< 0.001	24.77	12.79–47.94	0.031	0.033	0.001–0.738	0.02
Unavailability of the correct size of oropharyngeal airway	< 0.001	83.28	37.81–183.40	0.042	6.138	1.069–35.232	0.03
**Unavailability of a supraglottic airway device**	< 0.001	62.17	31.37-123.19	**0.007**	9.154	1.841–45.520	**0.01**
**Unavailability of the correct size laryngoscope blade**	< 0.001	114.98	46.25-285.84	**< 0.001**	39.935	4.752-335.618	**0.01**
Unavailability of a stylet or a bougie for difficult intubation	< 0.001	48.92	24.96–95.88	0.345	2.263	0.415–12.332	0.04
Unavailability of ETCO_2_ monitoring	< 0.001	53.42	27.29-104.58	0.040	5.267	1.075–25.806	0.03
Discontinuation of CPR greater than 10 s	< 0.001	42.32	22.32–80.56	0.788	1.261	0.232–6.864	0.05
**Delay in loading medications**	< 0.001	38.73	18.89–79.38	**0.014**	13.535	1.683-108.864	**0.02**
Error in administering medications	< 0.001	99.21	29.88-329.37	0.409	3.0456	0.215–43.451	0.04
Lack of monitoring chest compression fraction(CCF)	< 0.001	57.55	29.53-112.16	0.523	1.549	0.405–5.932	0.05
Lack of CPR feedback monitoring device	< 0.001	19.91	11.33–34.99	0.055	4.00	0.970-16.505	0.04

Legend: CPR, Cardio-pulmonary resuscitation; CCF, Chest compression fraction; ETCO_2,_ End-Tidal Carbon Dioxide

**Fig. 2:**
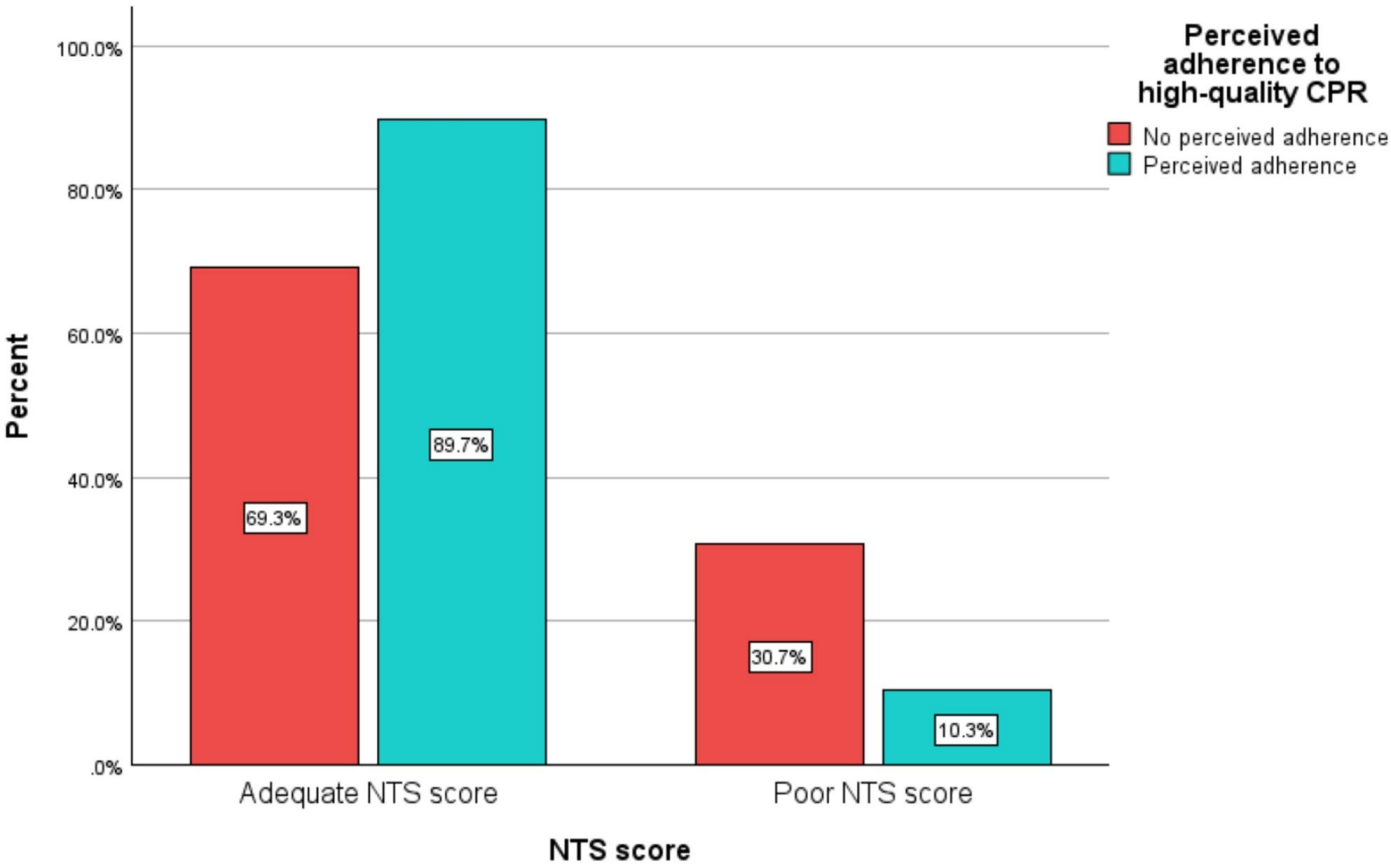
Bar diagram showing the association of poor non-technical skills with perceived non-adherence to high-quality CPR. Among the 68 participants with perceived adherence to high-quality CPR, 61 (89.7%) had adequate NTS, while only 10.3% did not. Among the 332 participants with perceived non-adherence to high-quality CPR, only 69.3% had adequate NTS, whereas 30.7% did not (Chi-Square p-value < 0.001)

The poor NTS score is significantly associated with a perceived non-adherence to high-quality CPR (Fig. 2).

Among the 68 participants with perceived adherence to high-quality CPR, 61 (about 90%) had adequate NTS, while only about 10% did not. Among the 332 participants with perceived non-adherence to high-quality CPR, only about 69% had adequate NTS, whereas 31% did not (Chi-Square p-value < 0.001). (Additional file 2, Table 11).

Regarding the technical domain, the total score across 17 items was 85, and a poor technical domain score was ≥ 50 points. The Chi-Square test showed that 15 items were significantly associated with a poor technical domain score. (Additional files 3). The univariate analysis of the poor technical domain score identified 15 items that were associated with it. The multivariable logistic regression analysis with a BH p-value correction revealed that only five items predicted a poor technical domain score (Table 4). The items were inability to identify cardiac rhythm (aOR 26.88, 95% CI 3.59-201.01, p-value < 0.001), non-working defibrillator (aOR 15.19, 95% CI 2.02–114.10, p-value 0.008), unavailability of supraglottic-airway device (SAD) (aOR 9.15, 95% CI 1.84–45.52, p-value 0.007), unavailability of the correct size laryngoscope blade (aOR 39.93, 95% CI 4.75-335.61, p-value < 0.001), and a delay in loading medications (aOR 13.53, 95% CI 1.68-108.86, p-value 0.0014). We also performed a role-based analysis of the technical domain barrier (unavailability of SAD) to predict a poor technical domain score. Upon excluding respiratory therapists, the aOR for the unavailability of SAD predicting a poor technical domain score remained statistically significant, but it was similar to that for the IP members taken together (aOR = 9.059; 95% CI: 1.129–72.685; *p* = 0.038). However, when we removed the doctor subgroup, the aOR increased to 10.59, though the p-value was marginally greater than 0.05 ( p-value 0.07).

Regarding technical domain barriers, among the 68 participants with perceived adherence to high-quality CPR, 63 (about 93%) had no technical domain barriers, while only 7% had such barriers. Among the 332 participants with perceived non-adherence to high-quality CPR, about 69.9% had no technical domain barriers, whereas about 30% had technical domain barriers (Chi-square p-value < 0.001). (Additional file 3, Table 29).

**Fig. 3:**
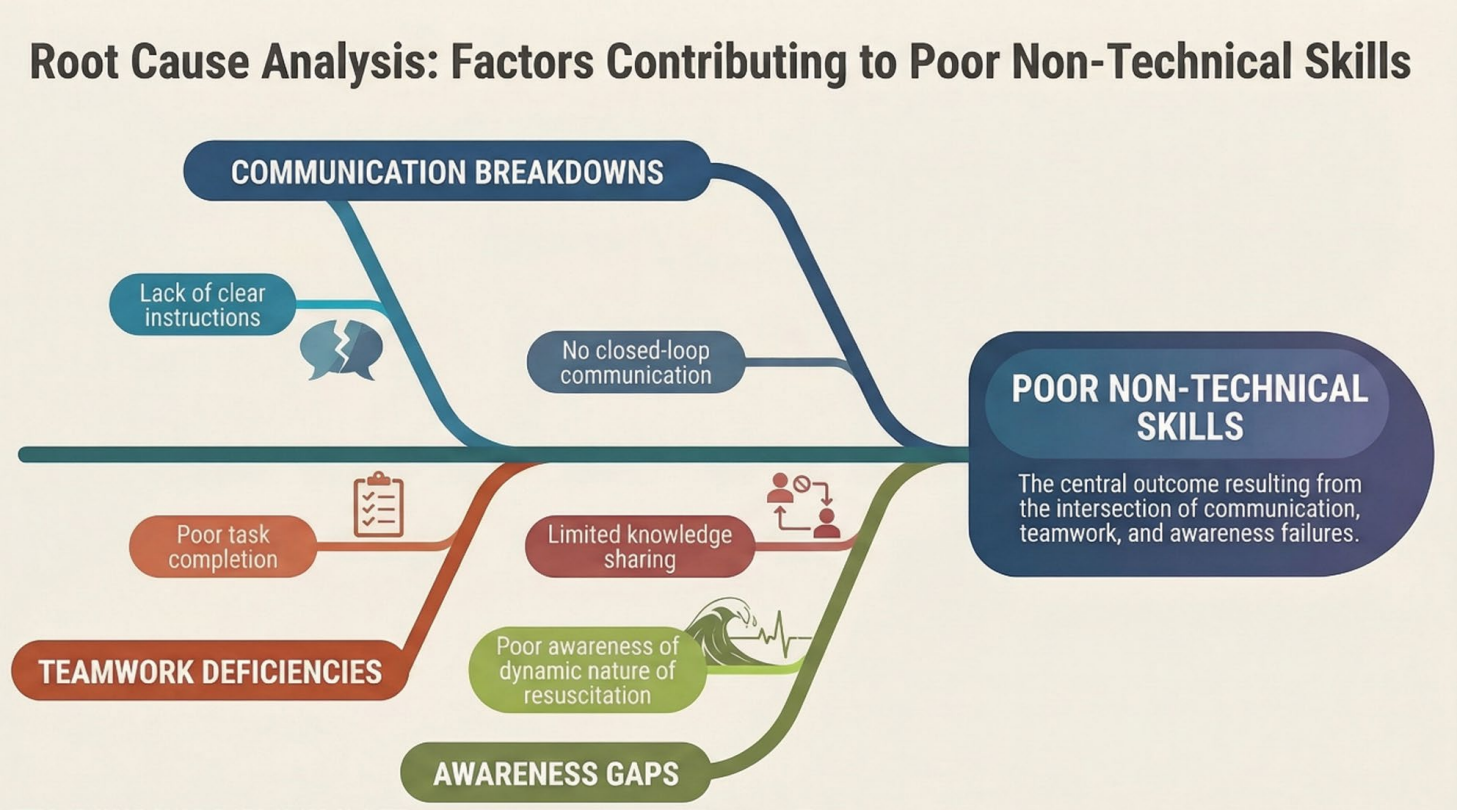
The Ishikawa diagram to visually map the NTS domain barriers, which independently predicted poor NTS scores. The authors used Google NotebookLM to generate the infographic in Fig. 3, based on concepts developed by the authors. All outputs were reviewed and verified by the authors

An Ishikawa diagram was made to visually map the NTS domain barriers, which independently predicted poor NTS scores (Fig. 3).

## Discussion

This survey investigated the perceived technical barriers and NTS barriers encountered by IP team members during IHCA scenarios. We found that the NTS domain barriers that independently predicted poor NTS scores were a lack of clear instructions during CPR by the team leader; a lack of awareness of the dynamic nature of resuscitation among the IP team members; a lack of closed-loop communication; and a lack of task completion and knowledge sharing. The technical domain barriers that independently predicted poor technical domain scores were a lack of ECG rhythm identification by the IP team members, a non-working defibrillator, unavailability of a SAD, a laryngoscope blade of the incorrect size during airway management, and a delay in loading medications prior to administration during CPR. Both poor NTS and poor technical domain scores were associated with incomplete adherence to AHA CPR guidelines during a CPR scenario.

The study by Ornato JP et al. provides real-world evidence from a large national registry demonstrating that technical domain barriers, including delays in defibrillation, medication administration deficiencies, and airway management issues, are significantly associated with decreased chances of return of spontaneous circulation [24]. NTS barriers, such as delays in identifying the team leader and in code team oversight, also contribute to resuscitation errors [24]. These findings are consistent with ours, in which both the technical domain and NTS barriers were significantly associated with perceived non-adherence to high-quality CPR.

Lack of clear instructions and closed-loop communication diminishes CPR quality. Evidence from resuscitation and human factors research demonstrates that ineffective communication, including the absence of clear instructions and a lack of closed-loop communication, is associated with poorer team performance and lower CPR quality. Studies have shown that structured communication strategies, including closed-loop communication, improve task completion, reduce errors, and enhance adherence to resuscitation algorithms during CPR scenarios [25].

Present evidence-based tools (Team Strategies and Tools to enhance Performance and Patient Safety, or Team STEPPS) provide specific strategies to improve communication during CPR, scenario-based instruction on team leadership, closed-loop communication, role clarity, and coordination during emergencies [26, 27]. A recent study, however, showed that using consensus-based standardised “action-linked communication” reduces chest compression pauses during defibrillation, airway management like intubation, and rhythm checks, without increasing mental workload, frustration index, or mental demand compared to closed-loop communication [27, 28]. While closed-loop communication may be advantageous for tasks such as medication preparation, standard communication is appropriate for time-critical actions, such as initiating chest compressions, where eliminating repeated confirmation phrases can preserve valuable seconds [27]. The NTS domains that were independent predictors of a poor NTS score in our study were similar to a web-based structured questionnaire study on barriers during CPR, where the authors found a lack of speech clarity in a crowded area during CPR, and a lack of collaboration between the IP members and failed closed-loop communication [29]. In another study on hot debriefings after in-hospital cardiac arrests during paediatric IHCAs, the most frequent negative comments involved lack of co-operation and coordination [30].

Another aspect of NTS that we found to be a barrier is a lack of understanding of the dynamic nature of resuscitation. Our findings were consistent with prior literature, which concluded that situational awareness is an important human factor that can be improved during CPR [31, 32]. The CPR quality bundle also highlights that dynamic, random teams and the dynamic nature of resuscitation should work harmoniously to improve resuscitation [33].

CPR performance is often disrupted when rescuers attempt to perform multiple tasks simultaneously, leading to workflow fragmentation [34, 35]. Among all NTS parameters, the lack of task completion assigned to a team member was the strongest predictor of a poor NTS score in our study. In a trial comparing CPR guideline adherence between ad hoc resuscitation teams and preformed resuscitation teams that had completed structured team building before cardiac arrest scenarios, the results demonstrated that ad hoc teams performed significantly worse, with reduced hands-on chest compression time and notable delays in both first defibrillation and epinephrine administration [36]. These findings highlight the importance of predefined team roles and prompt the implementation of tasks as soon as resuscitation begins [36]. The results emphasize that reliable and timely task execution is fundamental to high-quality resuscitation. Pre-formed CPR teams may benefit from predefined structures and task assignments, which can help complete essential actions more efficiently.

In our survey, we found the major technical domain barriers to be inadequate equipment availability, gaps in airway management, and delays in rhythm recognition. A qualitative study by M. Janatolmakan et al. also identified barriers related to the unavailability of equipment, including bag-valve-mask (BVM), laryngoscopes, and ventilators [37]. Defibrillator unavailability or a non-working defibrillator was the major critical challenge, which led to resuscitation failure. Monitoring of all CPR equipment and reporting the deficiencies and shortcomings to the CPR team by a team member was suggested [37].

Our findings identified delayed rhythm recognition as a technical barrier during resuscitation. In line with this, a study by NG J showed that though nurses can easily identify recognizable rhythms (such as ventricular tachycardia), they have difficulty detecting subtle changes or electrolyte-induced abnormalities [38].

Hence, training that emphasizes dynamic ECG interpretation and decision-making during simulated training rather than static ECG pattern recognition alone is important [38].

We also found that the unavailability of an SAD was a technical barrier, possibly because early insertion of an SAD results in a significantly higher chest compression fraction, better ventilation parameters, and fewer interruptions between compressions [39].

We found an increase in the aOR for SAD unavailability as a predictor of a poor technical domain score. In the context of Indian healthcare environments, doctors, particularly critical care and emergency physicians, are primarily responsible for airway management, including intubation during in-hospital cardiac arrest scenarios.

Thus, their exclusion may have increased the perceived adjusted odds of barrier among non-physician respondents who are less directly involved in airway interventions. Conversely, respiratory therapists in many Indian centres may not routinely perform intubation independently, which could explain why their exclusion did not lead to a similar change in effect size.

These findings suggest that perceptions of airway equipment barriers are broadly distributed across the IP team and are shaped in part by role-specific responsibilities and training patterns.

To address the challenges encountered during resuscitation, integrated strategies combining simulation-based training replicating real-time complexities with improvements in resource accessibility and team preparedness are essential.

## Clinical implications

The identification of technical domains and NTS barriers to perceived non-adherence to high-quality CPR in this study has direct practical relevance for improving resuscitation performance in hospital settings. Technical domain barriers, such as unavailability or malfunction of essential equipment, may highlight the need for regular equipment audits, standardized crash cart checks, and periodic skill-based refresher training for IP team members involved in CPR. At the same time, NTS barriers, including challenges in communication, leadership, and team coordination during resuscitation events, highlight the importance of incorporating structured team-based simulation training and crisis resource management principles into resuscitation education programs. Evidence suggests that improving both technical competence and NTS can significantly enhance adherence to high-quality CPR [8, 25]. Additionally, recognizing barriers enables healthcare institutions to identify units or teams that require targeted educational interventions, focused simulation exercises, or system-level quality improvement initiatives. Incorporating these findings into hospital resuscitation committees, training curricula, and continuous quality improvement programs may help improve team performance during CPR and ultimately improve patient outcomes [40].

By integrating perception-based barrier assessment, Likert-based scoring, and regression modelling, this study addresses a gap in identifying independent predictors of poor NTS and technical domain scores and provides a clearer analysis of whether NTS domain variables also influence adherence to AHA resuscitation guidelines. The findings directly support the AHA 2025 emphasis on strengthening NTS, such as leadership, communication, teamwork, and situational awareness.

This study has several limitations that should be considered while interpreting the findings. First, the study employed a convenience sampling strategy, which may limit the representativeness of the study population and introduce potential sampling bias.

Second, participation in the survey was voluntary, and therefore, self-selection bias cannot be excluded, as individuals with greater interest, awareness, or experience in resuscitation practices may have been more inclined to respond. Another limitation of this study is the potential for social desirability bias. Further, health-care personnel in remote areas may not respond to the questionnaires, leading to non-response bias.

Third, the findings of this study should be interpreted with appropriate caution, given the study design and data source. As the analysis was based on a perception-based survey, the identified associations between technical domain barrier and NTS barrier scores and perceived non-adherence to high-quality CPR reflect participants’ perceived experiences rather than objectively measured resuscitation performance. The findings are based on self-reported perceptions of barriers to high-quality CPR rather than direct observation of resuscitation performance, which may be subject to reporting bias or recall bias.

Finally, the study was conducted within the Indian healthcare context, and differences in healthcare infrastructure, training systems, and institutional protocols across regions may limit the external validity and generalizability of the findings to other healthcare settings. Future studies using multicentre sampling across diverse healthcare systems and incorporating objective assessments of resuscitation performance may further strengthen the evidence base.

## Conclusion

Key barriers contributing to poor NTS performance included unclear leadership instructions, inadequate adaptation to the dynamic nature of resuscitation, absence of closed-loop communication, incomplete task execution, and limited knowledge sharing within the team. Poor NTS scores were associated with non-adherence to high-quality CPR. In the technical domain, non-functional defibrillators, a lack of appropriate airway devices, and delays in medication preparation independently predicted poor TS performance. In the future, an NTS training protocol should be incorporated into CPR education and reinforced to enhance NTS performance.

## Supplementary Information

Below is the link to the electronic supplementary material.

Supplementary Material 1

Supplementary Material 2

Supplementary Material 3

## Data Availability

The datasets used and/or analysed during the current study are available from the corresponding author on reasonable request.

## Declarations

### Use of Artificial Intelligence (AI) tools for figure

The authors used Google NotebookLM to generate the infographic in Figs. 1 and 3 based on author-developed concepts. All outputs were reviewed and verified by the authors.

## Abbreviations

IHCA: In-hospital cardiac arrest
AHA: American Heart Association
CPR: Cardio-pulmonary resuscitation
NTS: Non-technical skills
IP: Interprofessional
CVR: Content validity ratio
CI: Confidence interval
VIF: Variance inflation factor
BH critical value: Benjamini–Hochberg critical value
SAD: Supraglottic airway device
AUROC: Area under the receiver operating characteristic curve
OR: Odd’s ratio
