# Supplementary material for: Unravelling technical domain barriers and non-technical skill barriers among interprofessional teams during in-hospital cardiac arrest: a questionnaire-based survey
Source: Int J Emerg Med. 2026 Apr 13;19:97. doi: 10.1186/s12245-026-01224-y (PMC13077877; doi:10.1186/s12245-026-01224-y)
Supplement: Supplementary file 1 — Supplementary Material 1 [file 12245_2026_1224_MOESM1_ESM.docx]

**Questions in the technical domain**

1. Have you experienced a delay in the initiation of CPR in wards of the hospital?
2. Have you experienced that vomitus/secretions/blood present on the patient delaying the initiation of CPR?
3. Have you experienced an inability to identify a particular cardiac rhythm as shockable or non-shockable as a barrier during CPR?
4. Have you experienced a non-working defibrillator as a barrier during CPR?
5. Have you experienced an unfamiliarity with the use of a different model defibrillator as a barrier during CPR
6. Have you experienced malfunctioning of the suction apparatus as a barrier during CPR?
7. Have you experienced the unavailability of an AMBU bag as a barrier during CPR?
8. Have you experienced the unavailability of the oropharyngeal airway of the correct size as a barrier during CPR?
9. Have you experienced the unavailability of a supraglottic airway device as a barrier during CPR?
10. : Have you experienced the unavailability of a correctly sized laryngoscope blade as a barrier during CPR?
11. Have you experienced a difficult intubation scenario during CPR due to the unavailability of a stylet or a bougie?
12. : Have you experienced the unavailability of an end tidal carbon dioxide (ETCO2) monitoring as a barrier during CPR?
13. : Have you experienced the discontinuation of CPR for >10 seconds to secure an advanced airway as a barrier during CPR?
14. Have you experienced a delay in loading medications as a barrier during CPR?
15. Have you experienced an error in the administration of medications as a barrier during CPR?
16. Have you experienced that the lack of monitoring of chest compression fraction (CCF) is a barrier during CPR?
17. Have you experienced a lack of a CPR feedback monitoring device as a barrier to high-quality CPR**?**

**Questions in the non-technical skills domain**

1. Have you experienced that the role allocation by the team leader during a CPR scenario was not done?
2. Have you experienced that there was a lack of clear instructions from the team leader during CPR?
3. Have you experienced a lack of awareness of the dynamic nature of the resuscitation scenario among the team members during CPR?
4. While performing CPR, did your focused attention get diverted due to other cases?
5. Have you experienced a lack of closed-loop communication among the team members as a barrier during CPR?
6. Have you faced any problems in critical decision-making from a team leader during CPR?
7. Have you experienced a lack of clear vocal summarization of the scenario during CPR as a barrier during resuscitation?
8. Have you experienced a lack of task completion assigned to a team member as a barrier during CPR?
9. Have you experienced a lack of knowledge sharing among team members as a barrier during CPR?

**Questions in the Logistics domain**

1. Have you experienced that there were NO "AHA ACLS trained" team members during CPR?
2. Do you participate in a post-resuscitation debriefing after completing CPR?
3. Have you experienced difficulty in reaching the location in your hospital where CPR is to be provided as a barrier during resuscitation?
4. Have you experienced overcrowding of patient relatives around the patient as a barrier during CPR?
5. In your experience, how frequently were you able to adhere to all the points as outlined below during a CPR scenario in your healthcare setup?
6. High-quality CPR, as per AHA protocol, includes the following points: a. Start compressions within 10 seconds after recognising cardiac arrest b. Chest compression rate – 100-120 compressions per minute c. Chest compression depth: At least 5cm for adults d. Chest compression fraction >80% e. Allow the chest to completely recoil after each compression f. Minimize interruptions in compressions to < 10 sec g. Give effective breaths to ensure a visible chest rise
