# Supplementary material for: Unravelling technical domain barriers and non-technical skill barriers among interprofessional teams during in-hospital cardiac arrest: a questionnaire-based survey
Source: Int J Emerg Med. 2026 Apr 13;19:97. doi: 10.1186/s12245-026-01224-y (PMC13077877; doi:10.1186/s12245-026-01224-y)
Supplement: Supplementary file 2 — Supplementary Material 2 [file 12245_2026_1224_MOESM2_ESM.docx]

**Chi-square test for non-technical skill (NTS) domain with Poor NTS score**

Table 2: Association between frequency of lack of role allocation by team leader and poor NTS score

| Lack of role allocation by the team leader | Adequate NTS score (<75^th^ percentile) | Poor NTS Score  (≥75^th^ percentile) | Chi- Square test p-value |
| --- | --- | --- | --- |
| Never/rarely/sometimes | 267 (91.8%) | 23 (21.1%) | P <0.001 |
| Often/very often | 24 (8.2%) | 86 (78.9%) |  |
| Total | 291 (100%) | 1. 00%) |  |

Table 3: Association between the frequency of lack of clear instructions by the team leader and poor NTS score

| Lack of clear instructions by the team leader | Adequate NTS score  (<75th percentile) | Poor NTS Score  (≥75th percentile) | Chi- Square test p-value |
| --- | --- | --- | --- |
| Never/rarely/sometimes | 283 (97.3%) | 26 (23.9%) | p <0.001 |
| Often/very often | 8 (2.7%) | 83 (76.1%) |  |
| Total | 291 (100%) | 1. 00%) |  |

Table 4: Association between the frequency of lack of awareness of the dynamic nature of resuscitation among team members and poor NTS score

| Lack of awareness of the dynamic nature of resuscitation among team members | Adequate NTS score  (<75th percentile) | Poor NTS Score  (≥75th percentile) | Chi- Square test p-value |
| --- | --- | --- | --- |
| Never/rarely/sometimes | 283 (97.3%) | 23 (21.1%) | p <0.001 |
| Often/very often | 8 (2.7%) | 86 (78.9%) |  |
| Total | 291 (100%) | 109 (100%) |  |

Table 5: Association between frequency of lack of focused attention due to other cases among team members and poor NTS score

| Focused attention diversion due to other cases | Adequate NTS score  (<75th percentile) | Poor NTS Score  (≥75th percentile) | Chi- Square test p-value |
| --- | --- | --- | --- |
| Never/rarely/sometimes | 285 (97.9%) | 48 (44.0%) | p <0.001 |
| Often/very often | 6 (2.1%) | 61 (56.0%) |  |
| Total | 291 (100%) | 109 (100%) |  |

Table 6: Association between frequency of lack of closed-loop communication among the team members and poor NTS score

| Lack of closed-loop communication among the team members | Adequate NTS score  (<75th percentile) | Poor NTS Score  (≥75th percentile) | Chi- Square test p-value |
| --- | --- | --- | --- |
| Never/rarely/sometimes | 279 (95.9%) | 21 (19.3%) | p <0.001 |
| Often/very often | 12 (4.1%) | 88 (80.7%) |  |
| Total | 291(100.0%) | 109 (100.0%) |  |

Table 7: Association between frequency of lack of critical decision-making by the team leader and poor NTS score

| lack of critical decision-making by the team leader | Adequate NTS score  (<75th percentile) | Poor NTS Score  (≥75th percentile) | Chi- Square test p-value |
| --- | --- | --- | --- |
| Never/rarely/sometimes | 287 (98.6%) | 43 (39.4%) | p <0.001 |
| Often/very often | 4 (1.4%) | 66 (60.6%) |  |
| Total | 291 (100%) | 109 (100%) |  |

Table 8: Association between the frequency of lack of clear vocal summarization of the scenario among team members and poor NTS score

| Lack of clear vocal summarization of the scenario | Adequate NTS score  (<75th percentile) | Poor NTS Score  (≥75th percentile) | Chi- Square test p-value |
| --- | --- | --- | --- |
| Never/rarely/sometimes | 283 (97.3%) | 28 (25.7%) | p <0.001 |
| Often/very often | 8 (2.7%) | 81 (74.3%) |  |
| Total | 291 (100%) | 1. 00%) |  |

Table 9: Association between the frequency of lack of task completion assigned to a team member and poor NTS score

| Lack of task completion assigned to a team member | Adequate NTS score  (<75th percentile) | Poor NTS Score  (≥75th percentile) | Chi- Square test p-value |
| --- | --- | --- | --- |
| Never/rarely/sometimes | 290 (99.7%) | 38 (34.9%) | p <0.001 |
| Often/very often | 1 (0.3%) | 71 (65.1%) |  |
| Total | 291(100%) | 109 (100%) |  |

Table 10: Association between the frequency of lack of knowledge sharing among team members and poor NTS score

| lack of knowledge sharing among team members | Adequate NTS score  (<75th percentile) | Poor NTS Score  (≥75th percentile) | Chi- Square test p-value |
| --- | --- | --- | --- |
| Never/rarely/sometimes | 284 (97.6%) | 24 (22.0%) | p <0.001 |
| Often/very often | 7 (2.4%) | 85 (78.0%) |  |
| Total | 291(100%) | 109 (100%) |  |

**Table 11: Association between poor NTS score and perceived non-adherence to high-quality CPR**

| Non-technical skill (NTS) score | Perceived  non-adherence to  high-quality CPR | Perceived adherence  to high-quality CPR | Chi-square test p value |
| --- | --- | --- | --- |
| Adequate NTS score | 230 (69.3%) | 61 (89.7%) | p <0.001 |
| Poor NTS score | 102 (30.7%) | 7 (10.3%) |  |
| Total | 332 (100%) | 68 (100%) |  |

*High-quality CPR, as per the 2020 AHA protocol, includes the following components as listed below (a-g). Thus, perceived adherence to high-quality CPR refers to the perceived adherence to all the components listed below. Perceived non-adherence to high-quality CPR refers to omitting any one of the following components. These components were mentioned in the questionnaire survey and are the following:

a. Start compressions within 10 seconds after recognising cardiac arrest

b. Chest compression rate – 100-120 compressions per minute

c. Chest compression depth: At least 5cm for adults

d. Chest compression fraction >80%

e. Allow the chest to completely recoil after each compression

f. Minimize interruptions in compressions to < 10 sec

g. Give effective breaths to ensure a visible chest rise
