# Supplementary material for: Unravelling technical domain barriers and non-technical skill barriers among interprofessional teams during in-hospital cardiac arrest: a questionnaire-based survey
Source: Int J Emerg Med. 2026 Apr 13;19:97. doi: 10.1186/s12245-026-01224-y (PMC13077877; doi:10.1186/s12245-026-01224-y)
Supplement: Supplementary file 3 — Supplementary Material 3 [file 12245_2026_1224_MOESM3_ESM.docx]

**Chi-square test for technical domain with poor technical domain score**

**Table 12: Association between problems faced due to delay in initiation of CPR and poor technical domain score**

| Delay in initiation of CPR | Adequate technical domain score  (<75^th^ percentile) | Poor technical domain score  (≥ 75^th^ percentile) | Chi-Square test p-value |
| --- | --- | --- | --- |
| Never/rarely/sometimes s | 232 (78.6%) | 86(81.9%) | 0.477 |
| Often /very often | 63(21.4%) | 19 (18.1%) |  |
| Total | 295 (100%) | 105(100%) |  |

**Table 13: Association between problems faced due to Vomitus /secretions present on patients delaying the initiation of CPR and poor technical domain score**

| Presence of vomitus/secretions delaying CPR | Adequate technical domain score  (<75^th^ percentile) | Poor technical domain score  (≥ 75^th^ percentile) | Chi-Square test p-value |
| --- | --- | --- | --- |
| Never/rarely/sometimes s | 203 (68.8%) | 74 (70.5%) | 0.751 |
| Often /very often | 92 (31.2%) | 31 (29.5%) |  |
| Total | 295 (100%) | 105 (100%) |  |

Table 14: Association between problems faced due to inability to identify particular cardiac rhythm and poor technical domain score

| Inability to identify cardiac rhythm | Adequate technical domain score  (<75^th^ percentile) | Poor technical domain score  (≥ 75^th^ percentile) | Chi-Square test p-value |
| --- | --- | --- | --- |
| Never/rarely/sometimes s | 288(97.6%) | 43 (41%) | <0.001 |
| Often /very often | 7 (2.4%) | 62 (59 %) |  |
| Total | 295 (100%) | 105 (100%) |  |

**Table 15: Association between problems faced due to non-working defibrillator and poor technical domain score**

| Non-working defibrillator | Adequate technical domain score  (<75^th^ percentile) | Poor technical domain score  (≥ 75^th^ percentile) | Chi-Square test p-value |
| --- | --- | --- | --- |
| Never/rarely/sometimes | 285 (96.6%) | 43 (41%) | <0.001 |
| Often /very often | 10 (3.4%) | 62 (59%) |  |
| Total | 295 (100%) | 105 (100%) |  |

**Table 16: Association between problems faced due to unfamiliarity with different models of defibrillators and poor technical domain score**

| Unfamiliarity with different models of defibrillators | Adequate technical domain score (< 75th percentile) | Poor technical domain score  (≥ 75^th^ percentile) | Chi-Square test p-value |
| --- | --- | --- | --- |
| Never/rarely/sometimes s | 287(97.3%) | 34 (32.4%) | <0.001 |
| Often /very often | 8 (2.7%) | 71 (67.6%) |  |
| Total | 295 (100%) | 105 (100%) |  |

**Table 17: Association between problems faced due to malfunctioning of the suction apparatus and poor technical domain score**

| Malfunctioning of the suction apparatus | Adequate technical domain score (< 75th percentile) | Poor technical domain score  (≥ 75^th^ percentile) | Chi-Square test p-value |
| --- | --- | --- | --- |
| Never/rarely/sometimes s | 225(76.3%) | 25 (23.8%) | <0.001 |
| Often /very often | 70(23.7%) | 80 (76.2%) |  |
| Total | 295 (100%) | 1. 00%) |  |

**Table 18: Association between problems faced due to the unavailability of a bag-valve-mask (BVM) and poor technical domain score**

| Unavailability of an AMBU bag | Adequate technical domain score (< 75th percentile) | Poor technical domain score  (≥ 75^th^ percentile) | Chi-Square test p-value |
| --- | --- | --- | --- |
| Never/rarely/sometimes s | 281 (95.3%) | 47 (44.8%) | <0.001 |
| Often /very often | 14 (4.7%) | 58 (55.2%) |  |
| Total | 295 (100%) | 1. 00%) |  |

**Table 19: Association between problems faced due to unavailability of correct size oropharyngeal airway and poor technical domain score**

| Unavailability of the correct size oropharyngeal airway | Adequate technical domain score (< 75th percentile) | Poor technical domain score  (≥ 75^th^ percentile) | Chi-Square test p-value |
| --- | --- | --- | --- |
| Never/rarely/sometimes s | 286 (96.9%) | 29 (27.6%) | <0.001 |
| Often /very often | 9 (3.1%) | 76 (72.4%) |  |
| Total | 295 (100%) | 105 (100%) |  |

**Table 20: Association between problems faced due to unavailability of a supraglottic airway device and poor technical domain score**

| Unavailability of a supraglottic airway device | Adequate technical domain score, not poor(< 75th percentile) | Poor technical domain score  (≥ 75^th^ percentile) | Chi-Square test p-value |
| --- | --- | --- | --- |
| Never/rarely/sometimes s | 279 (94.6%) | 23 (21.9%) | <0.001 |
| Often /very often | 16 (5.4%) | 82 (78.1%) |  |
| Total | 295 (100%) | 105 (100%) |  |

**Table 21: Association between problems faced due to unavailability of the correct size laryngoscope blade and poor technical domain score**

| Unavailability of the correct size laryngoscope blade | Adequate technical domain score (< 75th percentile) | Poor technical domain score  (≥ 75^th^ percentile) | Chi-Square test p-value |
| --- | --- | --- | --- |
| Never/rarely/sometimes s | 289 (98%) | 31 (29.5%) | <0.001 |
| Often /very often | 6 (2%) | 74 (70.5%) |  |
| Total | 295 (100%) | 105 (100%) |  |

**Table 22: Association between problems faced due to unavailability of a stylet or a Bougie for a difficult intubation scenario and a poor technical domain score**

| Unavailability of a stylet or a bougie | Adequate technical domain score (< 75th percentile) | Poor technical domain score  (≥ 75^th^ percentile) | Chi-Square test p-value |
| --- | --- | --- | --- |
| Never/rarely/sometimes s | 280 (94.9%) | 29 (27.6%) | <0.001 |
| Often /very often | 15 (5.1%) | 76 (72.4%) |  |
| Total | 295 (100%) | 105 (100%) |  |

**Table 23: Association between problems faced due to the unavailability of ETCO_2_ monitoring and poor technical domain score**

| Unavailability of ETCO_2_ monitoring | Adequate technical domain score (< 75th percentile) | Poor technical domain score  (≥ 75^th^ percentile) | Chi-Square test p-value |
| --- | --- | --- | --- |
| Never/rarely/sometimes s | 263 (89.2%) | 14 (13.3%) | <0.001 |
| Often /very often | 32 (10.8%) | 91 (86.7%) |  |
| Total | 295 (100%) | 105 (100%) |  |

**Table 24: Association between problems faced due to discontinuation of CPR greater than 10 seconds and poor technical domain score**

| Discontinuation of CPR greater than 10 seconds | Adequate technical domain score (< 75th percentile) | Poor technical domain score  (≥ 75^th^ percentile) | Chi-Square test p-value |
| --- | --- | --- | --- |
| Never/rarely/sometimes s | 277 (93.9%) | 28 (26.7%) | <0.001 |
| Often /very often | 18 (6.1%) | 77 (73.3%) |  |
| Total | 295 (100%) | 105 (100%) |  |

**Table 25: Association between problems faced due to delay in loading medications and poor technical domain score**

| **Delay in loading medications** | Adequate technical domain score (< 75th percentile) | Poor technical domain score  (≥ 75^th^ percentile) | Chi-Square test p-value |
| --- | --- | --- | --- |
| Never/rarely/sometimes s | 284 (96.3%) | 42 (40%) | <0.001 |
| Often /very often | 11 (3.7%) | 63 (60%) |  |
| Total | 295 (100%) | 105 (100%) |  |

**Table 26: Association between problems faced due to errors in the administration of medications and poor technical domain score**

| Error in the administration of medications | Adequate technical domain score (< 75th percentile) | Poor technical domain score  (≥ 75^th^ percentile) | Chi-Square test p-value |
| --- | --- | --- | --- |
| Never/rarely/sometimes s | 292 (99%) | 52 (49.5%) | <0.001 |
| Often /very often | 3 (1%) | 53 (50.5%) |  |
| Total | 295 (100%) | 105 (100%) |  |

**Table 27: Association between problems faced due to lack of monitoring of chest compression fraction (CCF) and poor technical domain score**

| **Lack of monitoring of chest compression fraction (CCF)** | Adequate technical domain score (< 75th percentile) | Poor technical domain score  (≥ 75^th^ percentile) | Chi-Square test p-value |
| --- | --- | --- | --- |
| Never/rarely/sometimes s | 269 (91.2%) | 16 (15.2%) | <0.001 |
| Often /very often | 26 (8.8%) | 89 (84.8%) |  |
| Total | 295 (100%) | 105 (100%) |  |

**Table 28: Association between problems faced due to the lack of a CPR feedback monitoring device and poor technical domain score**

| Lack of a CPR feedback monitoring device | Adequate technical domain score (< 75th percentile) | Poor technical domain score  (≥ 75^th^ percentile) | Chi-Square test p-value |
| --- | --- | --- | --- |
| Never/rarely/sometimes s | 248 (84.1%) | 22 (21%) | <0.001 |
| Often /very often | 47 (15.9%) | 83 (79%) |  |
| Total | 295 (100%) | 105 (100%) |  |

**Table 29: Association between poor technical domain score and perceived non-adherence to high-quality CPR**

| Technical domain score | Perceived  non-adherence to  high-quality CPR | Perceived adherence  to high-quality CPR | Chi-square test p-value |
| --- | --- | --- | --- |
| Adequate technical domain score | 232 (69.9%) | 63 (92.6%) | p <0.001 |
| Poor technical  domain score | 100 (30.1%) | 5 (7.4%) |  |
| Total | 332 (100%) | 68 (100%) |  |
